# A short guide for medical professionals in the era of artificial intelligence

**DOI:** 10.1038/s41746-020-00333-z

**Published:** 2020-09-24

**Authors:** Bertalan Meskó, Marton Görög

**Affiliations:** 1 The Medical Futurist Institute, Budapest, Hungary; 2 Semmelweis University, Budapest, Hungary

**Keywords:** Medical research, Information technology, Communication

## Abstract

Artificial intelligence (A.I.) is expected to significantly influence the practice of medicine and the delivery of healthcare in the near future. While there are only a handful of practical examples for its medical use with enough evidence, hype and attention around the topic are significant. There are so many papers, conference talks, misleading news headlines and study interpretations that a short and visual guide any medical professional can refer back to in their professional life might be useful. For this, it is critical that physicians understand the basics of the technology so they can see beyond the hype, evaluate A.I.-based studies and clinical validation; as well as acknowledge the limitations and opportunities of A.I. This paper aims to serve as a short, visual and digestible repository of information and details every physician might need to know in the age of A.I. We describe the simple definition of A.I., its levels, its methods, the differences between the methods with medical examples, the potential benefits, dangers, challenges of A.I., as well as attempt to provide a futuristic vision about using it in an everyday medical practice.

## Introduction

**Fig. 1:**
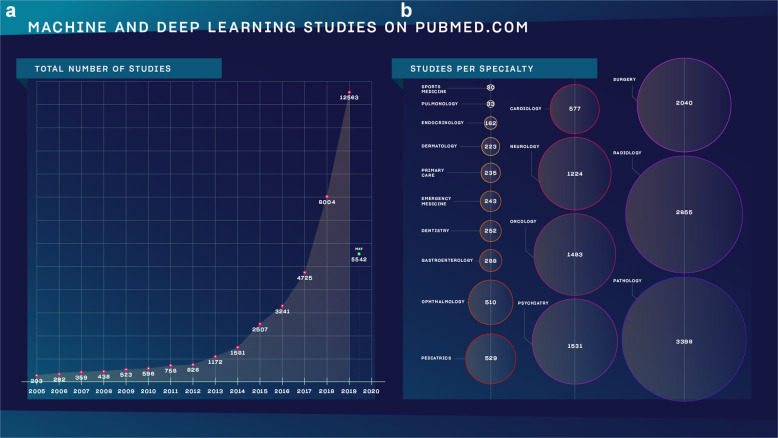
Number of medical A.I. studies by year from 2010 to 2020; and by medical specialties. **a** The number of studies as found on Pubmed.com using the search term)“machine learning” OR “deep learning”) and choosing a year in advanced search. **b** The same search method was used followed by (AND specialty) without specifying a time frame. The number in the circles determine how many studies we found.

Artificial intelligence (A.I.) is expected to significantly influence the practice of medicine and the delivery of healthcare in the near future. While there are only a handful of practical examples for its medical use with enough evidence, hype around the topic is unprecedented^1^. There is a growing list of publications on the subject in the form of academic articles, health policy reports, statements from professional societies, and popular media coverage (Fig. 1).

A.I. has been used extensively in industries such as transportation, entertainment or IT during the last decade. It has been used to control self-driving vehicles; to trade on the stock market; social media platforms, web browsers and search engines. It is likely that the reader of this paper used some forms of A.I. today for more than an hour by using services such as Google Maps, Waze, Facebook, Instagram, LinkedIn or Google Search, among a myriad of others. It has potentials in medicine, drug design and healthcare, yet, the proof and evidence are yet to be convincing enough for the general public and the medical community to adopt the technology^2^.

The technology is still in its infancy and more studies are published each year than the year before. There are so many papers, conference talks, misleading news headlines and study interpretations that a short and visual guide any medical professional can refer back to in their professional life might be useful.

There is no doubt that A.I. will have a beneficial role in healthcare and can penetrate the boundaries of adoption only if medical professionals serve as knowledgeable and supportive guides and leaders in the process^3^.

For this, it is critical that physicians understand the basics of the technology so they can see beyond the hype, evaluate A.I.-based studies and clinical validation; as well as acknowledge the limitations and opportunities A.I. has. This paper aims to serve as a short, visual and digestible repository of information and details every physician might need to know in the age of A.I.

We describe the simple definition of A.I., its levels, its methods, the differences between the methods with medical examples, the potential benefits, dangers, challenges of A.I., as well as we attempt at providing a futuristic vision about using it in an everyday medical practice.

## Definition and levels of A.I.

A.I. is an interdisciplinary field spanning computer science, psychology, linguistics, and philosophy, among others. According to its simplest definition, artificial intelligence (A.I.) is intelligence demonstrated by machines. It is sometimes also described as “machines that mimic cognitive functions that humans associate with the human mind, such as learning and problem solving”^4^.

**Fig. 2:**
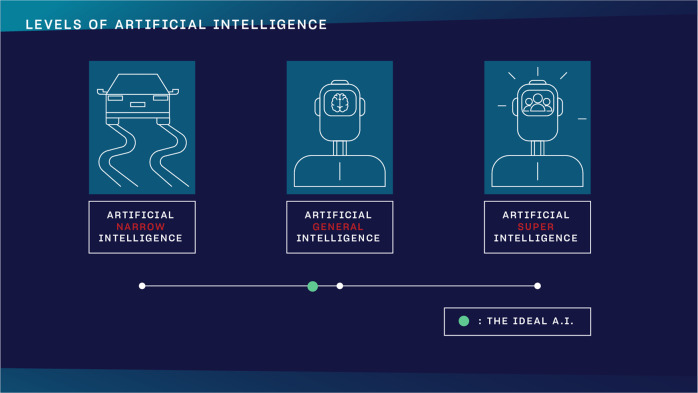
Levels of A.I. The three levels of A.I. as defined by Nick Bostrom in Superintelligence. The green dot indicates a theoretical threshold for what the ideal scenario would be.

Nick Bostrom, philosopher at the University of Oxford, described three major levels in the development of A.I. in his book Superintelligence (Fig. 2^5^).

### Artificial Narrow Intelligence (ANI)

ANI already has incredible pattern recognizing abilities in huge data sets, which makes it perfect for solving text, voice, or image-based classification and clustering problems. It is an algorithm that can excel at a precisely defined, single task. It can play chess like nobody else ever, yet its IQ is zero.

### Artificial General Intelligence (AGI)

one day could have a human being’s comprehensive and total cognitive capacity. This is human level A.I. It can reason, argue, memorize and solve issues like you do

### Artificial SuperIntelligence (ASI)

theoretically could have humanity’s combined cognitive capacity or even more. Humanity, obviously, would not be able to grasp its knowledge and understand its reasoning. Many organizations work hard to avoid ever reaching this stage.

## How does A.I. work?

A.I. works through a method called machine learning. As there are challenges and tasks so complicated in healthcare that writing traditional algorithms for solving those was not enough anymore, a new method was needed. Machine learning gives computers the ability to learn without being explicitly programmed^6^. If you feed the algorithm with enough data of good quality, machine learning allows them to create strategies for excelling at that particular task.

If I want to write a program that can spot cats on photos, I had better turn to machine learning. The reasons why quickly become clear if you try to devise rules that such a program should be based on. How can you spot a cat in a photo? If you devise features you think easily depict cats such as having two ears, two eyes, four legs and such, you find yourself in a situation where you also have to define all these expressions. What is an ear for a program that only “sees” pixels on a photo?

Therefore, the most efficient way is to feed a machine learning algorithm with images with cats on them, preferably with cats that have been annotated manually by human beings to make sure the images do contain cats. The more such annotated images we feed the algorithm with, the better it will become at recognizing cats on images. It will not understand what a cat is, but it will certainly recognize what we think are cats in photos like we do with an increasing and ruthless efficiency.

No matter what task we aim to solve, we feed the simpler machine learning algorithms with data and constantly iterate how it digests it to become better at solving the task. With more complex algorithms such as neural networks and deep learning, it is possible that the algorithm starts creating its own rules and strategies without human input. From there, not even its developers might understand how it draws a conclusion or the strategy it uses to excel at a task.

When it comes to technologies we use to make medical decisions, we like to understand the core of the machine or at least the physical/biological explanations behind it. In the case of advanced A.I.-based algorithms, it seems we will not be able to understand more than the theoretical basics.

## Real-life examples for the subtypes of machine learning

**Fig. 3:**
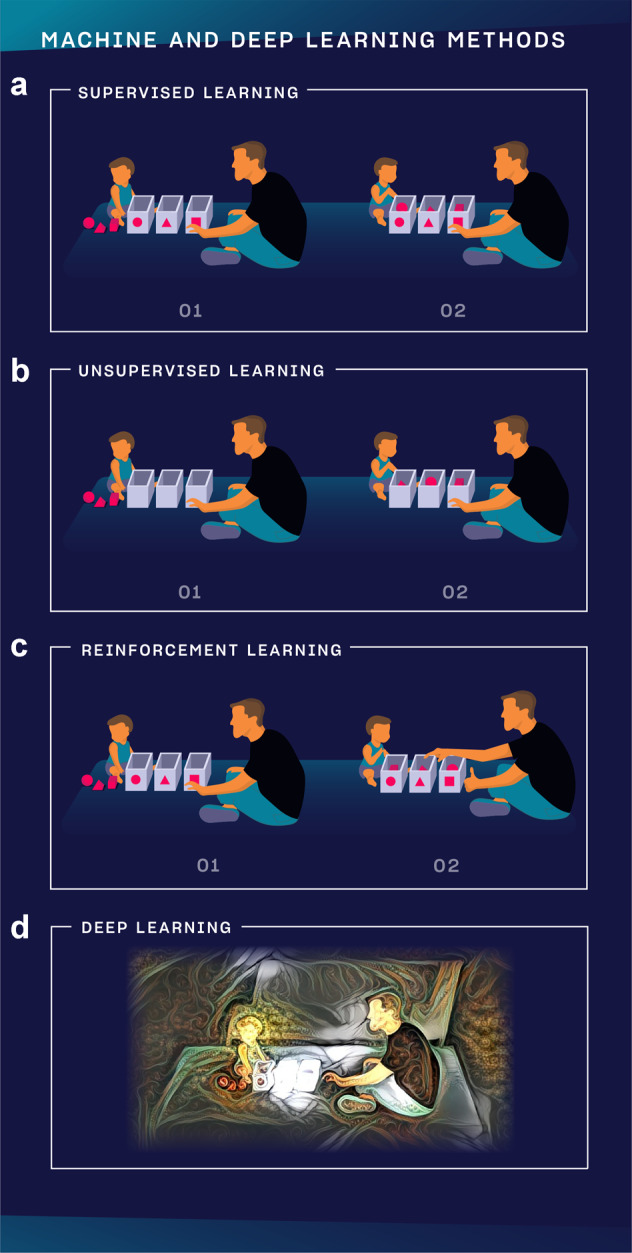
Visual guide to machine and deep learning subtypes. **a** In supervised learning, the teacher (developer) knows what he wants to teach to the child (A.I.), defines the expected answer and the child learns to excel at the task. **b** In unsupervised learning, the teacher does not influence how the child learns to play but observes the conclusions the child can draw from solving the task. **c** In reinforcement learning, the teacher knows what he wants to teach to the child but does not define step-by-step how the child should learn it. Instead, the teacher only gives feedback after the task is completed and asks the child to find out his own strategy using those outcomes the teacher rewarded. **d** In deep learning, it is possible to analyze vastly more complex data sets from images and videos to a sort of human reasoning. It is multi-layered and could mimic how neural networks in the brain work.

Machine learning has many subtypes and combined methods, but we only feature the three major subtypes besides an advanced method, deep learning (Fig. 3).

### Supervised learning

Is used when we can precisely define the task we want the algorithm to learn based on data that we already have. Let’s take the following example. We have two sets of medical records of patients, group A and B. In one set, we have family history, lab markers and other details with the diagnosis. In the other set, we have the same kinds of data but without the diagnosis. We would like to build a model that can learn to assign the right diagnosis to patients in group B based on the associations and labels the algorithm learns about in group A. It is like learning with a teacher because we know exactly what the algorithm should learn, and it is by far the most frequently used training mode.

### Unsupervised learning

Is like learning without a teacher. We have a group of patients with different sets of data, but we do not know their individual diagnoses. We build a model, then to try to cluster patients based on similar attributes such as the symptoms they presented with, their lab markers or age and gender. We might learn new associations we have not looked at before. In another example, it can also be useful in clustering tissue samples based on similar gene expression values; or in finding novel drug-drug interactions. In summary, we devise certain rules, let the algorithm learn by itself and we do not modify the algorithm based on the outcome.

### Reinforcement learning

Allows the algorithm to learn how to complete the tasks with a sequence of decisions by itself without being told how to do it. The teacher is only able to give feedback after a series of actions, not for each item as it does with supervised learning. The model starts performing the task only knowing some basic rules, and after failing or succeeding in completing the task, the teacher weighs in to push it to use the winning strategy more. This way, the program can build its own experiences as it performs the task more and more. It is similar to how we train dogs. When the dog performs or tries to perform a task, we only give it a treat if it performed well.

The most famous example for this method is how AlphaZero can learn to become the best player in any 2-players game in hours by playing millions of games against itself. It starts playing a game knowing only its basic rules, and the developers let the algorithm know when it won a match to prioritize that strategy while playing the next game^7^.

In an example, authors used this method to determine clinical trial dosing, where the algorithm learnt the appropriate dosing regimen to reduce mean tumor diameters in patients undergoing chemo- and radiation therapy^8^. The main challenge with applying reinforcement learning to healthcare is that we cannot play out a large number of scenarios as the lives of patients are at stake.

## Machine learning vs traditional statistical models

A.I. is a huge domain which includes machine learning, evolutionary algorithms, but also methods that do not even process quantitative data. Therefore, it makes sense to compare machine learning to traditional statistical methods.

Traditional statistical models focus on discovering relationships and confidence intervals between data points and outcomes. Compared to this, machine learning methods aim to reach high prediction accuracy with putting less emphasis on whether it is possible to interpret the model. The former often analyses a given dataset for its insights, while the latter is trained on a set of data, evaluated on a second set, and then used on an unseen third.

Thus, prediction is key in machine learning to generate otherwise unavailable (e.g., expensive or not yet known) data. Also, machine learning is often better suited for large numbers of input variables (think of images with thousands of pixels), while traditional analysis with statistical models was designed for data with tens of input columns^9^.

## Machine vs deep learning

Machine learning is a broad set of methods, their majority were used even decades before the current A.I. revolution. Most breakthroughs are achieved nowadays with artificial neural networks, but there are several other models, each with its own advantages.

Deep learning is a subset of machine learning and while it has similar functions, its capabilities are different. Deep learning uses a layered structure of artificial neural networks that is inspired by the neural network of the human brain. The internal structure and number of layers within a neural network is a field of active research, but as a rule of thumb we can say that a deeper net with more layers can learn more complex tasks - at the same time requiring more data and longer time to train. Deep models have the capacity to process images, sound and other high dimensionality data with good results, while other machine learning models may perform better with data organized into a spreadsheet.

Looking at a medical example, let’s build a model that can cluster patients by diagnosis based on the data in their medical records. If a medical record contains the expression Type 1 Diabetes, a machine learning model will learn to put all such patients in the Type 1 Diabetes cluster. But a deep learning algorithm could learn with time without human input that patients with medical records that only mention T1D should also be assigned to the same group. Programmers of other machine learning algorithms should add these alternatives themselves.

Another example might also demonstrate the power and potentials of deep learning. We would like to build a model that turns on the light if we shout the word dark. A deep learning model, by time, would learn that saying “I can’t see” or “it’s dark here” should also turn on the light.

## How to evaluate news and studies about A.I.?

Hardly a day goes by without promising research papers and studies on how to apply machine and deep learning to medical problems. However, as already just mentioning A.I. makes companies’ prospects better on any market, overhyping and overmarketing what an algorithm can do is an everyday phenomenon. There are still ways that help evaluate research papers and news on A.I. There are some general questions we can ask ourselves while reading medical A.I. papers. (Table 1)^10,11^.

**Table 1:** A summary of some general questions readers of an A.I. medical paper might want to ask themselves to evaluate the quality of the results of a research.

**Questions**
What aspects of existing clinical practice does this system reinforce?
Are the sizes of the training, validation, and test sets justified?
How can we be sure the training data matches what we expect to see in real life and does not contain bias?
How can we be confident of the quality of the ‘labels’ the system is trained on?
Was the A.I. algorithm trained using a standard of reference that is widely accepted in our field?
Was the manner in which the A.I. algorithm makes decisions demonstrated?
Were the results of the A.I. algorithm compared with experts in my field?
Is the system applied to the same diagnostic context that it was trained in?
Is the A.I. algorithm publicly available?

Besides obvious features such as the quality of the journal the paper was published in (real breakthroughs tend to be able to reach the attention of high-level journals), the most important factor is the source of data. It matters where the data stems from, thus it is worth checking the ‘Methods’ section where authors describe how, where and what kind of data they received. No algorithm can be trained without a good amount of quality data.

Moreover, the size of the dataset also matters: the more images, text or any other source materials the researchers have, the more precise the algorithms can become. Collaborations with clinicians and healthcare institutions are crucial in getting large amounts of quality data. If not, some research groups do tricks on their dataset to make it bigger (e.g., rotate images to double the database size).

The reported performance (e.g., precision, speed) of the algorithm should be compared to previously existing solutions and human abilities. Even a state-of-the-art technical solution may perform so much worse than human professionals in a real-world clinical setting, that it may not be helpful at all. It is crucial for such technologies to be evaluated whether they can easily be implemented in clinical protocols, and whether the results are straightforward for the medical staff to interpret.

It is also key to see whether the paper analyzed a real clinical problem. An algorithm might perform very well on a pre-selected dataset, however, it should also be tested on real clinical data. DeepMind claimed in its published study that its model was able to accurately predict that a patient will develop acute kidney failure “within a clinically actionable window” up to 48 h in advance^12^. While the algorithm did do that, it cannot be clinically validated without testing it prospectively in a clinical setting.

Also, when you look at the latest pieces of news on smart algorithms, you should watch out for the following to be able to carefully assess the quality of the given A.I.-related article. The term “artificial intelligence” itself might be misleading as due to the overuse of the expression, its meaning started to get inflated. If an announcement or news article mentions A.I. without describing the exact method under it, it is a good sign to be skeptical and careful. A company or a research group should mention a subtype of machine or deep learning and be able to explain the method in detail with which they are aiming to create A.I.

In a relevant example that took place in Thailand, a Google-backed algorithm aimed at improving care. There, normally, nurses took photos of the eyes of patients during check-ups and shared those to be analyzed by a specialist elsewhere. This process could take up to 10 weeks. The A.I. developed by Google Health identified signs of diabetic retinopathy from an eye scan with an accuracy of more than 90% and provide a result in less than 10 min. When Google started putting this system into practice, they ran into real-life issues. Sometimes a bad internet connection stopped the whole system from working as all images had to be uploaded to the cloud through a strong connection. If the quality of the scan did not meet a certain threshold, it simply did not give a result (the deep learning algorithm has to be fed with high quality images to get better). This way, nurses had to spend time editing some of the images the algorithm chose not to analyze. Developers had to travel to the site to help sort out these issues^13^.

As a summary, the medical journal Radiology proposed a guide for authors, reviewers, and readers for assessing radiology research on artificial intelligence^10^. Other medical associations and journal editorial boards might adopt and customize it too. They also describe a checklist that every author should complete when publishing their A.I. research.

## Examples for how healthcare could benefit from A.I.

In short, tasks that are highly repetitive and involve the analysis of quantifiable data might benefit the most from the use of A.I. We feature a few examples that provide a picture about the whole range of opportunities.

### Improving in-person and online consultations

Babylon Health launched an application that offers A.I.-driven consultation. It uses the patient’s medical history and common medical knowledge. Patients report their symptoms through the app which checks those in a database of diseases using speech recognition. After that, it offers a course of action^14^.

### Health assistance and medication management

The medical start-up Sense.ly developed Molly, a virtual nurse that was designed to have a smiling face coupled with a pleasant voice. Its goal is to help assist patients with monitoring their health or disease management in-between doctor’s visits using ML. It also provides customized follow-up care, with a focus on chronic diseases^15^. A similar approach was used by the AiCure app that uses a smartphone’s camera and A.I. to confirm that patients are adhering to their prescriptions^16^. This could be useful for people with serious medical conditions or participants in clinical trials.

### A.I.-driven diagnostics

In 2020, the FDA approved a software programme from the company Caption Health that allows medical professionals to perform cardiac ultrasound imaging without specialized training. It uses A.I. to provide real-time guidance and also the ability to save images of diagnostic quality. It acts as a “co-pilot” for those performing an ultrasound scan as it was designed to emulate the guidance that an expert sonographer would provide to optimize the image. It gives instructions on how to manipulate the transducer, and provides automated feedback on diagnostic image quality^17^.

### Mining medical records

Collecting, storing, normalizing, and tracking medical records is an obvious step for A.I. As an example, Google Deepmind Health is cooperating with the Moorfields Eye Hospital NHS Foundation Trust to improve eye treatment by analyzing retina scans. The images are analyzed by DeepMind’s algorithms resulting in a detailed diagnosis and a so-called “urgency score” in about 30 s. The prototype system can detect diabetic retinopathy, glaucoma, and age-related macular degeneration^18^.

### Precision medicine

The company Deep Genomics aims at identifying patterns in genetic data and medical records of patients trying to link mutations to medical conditions. Oncompass Medicine uses A.I.-based algorithms to match genetic mutations found in patients’ tumor samples with ongoing clinical trials worldwide. This way, patients can receive precisely targeted treatments specific to the kind of cancerous tissue they have.

### Designing treatment plans

IBM Watson developed a software that provides evidence-based treatment options for oncologists. It was designed to analyze both structured and unstructured data in medical records that may contribute to decision-making about treatment pathways. The software combines data from the patient’s medical record with clinical expertise, and research papers to suggest promising treatment plans^19^. There are many similar examples in other specialties. Creating an optimized radiation therapy delivery plan usually takes days. A.I.-based technologies help speed this process, completing the process in a couple of minutes^20,21^.

### Drug creation

The way pharmaceutical companies develop new drugs through clinical trials can take several years and can cost billions of dollars. Speeding this up while making it more cost-effective would have an enormous effect on healthcare. The company Atomwise uses supercomputers to root out treatments from a database of molecular structures. They also launched a search for a previously unknown combination of safe and existing medicines and in a few days found two drugs predicted by the company’s A.I. technology which may significantly reduce the infectivity of Ebola. Such analysis typically would have taken months or years^22^.

### Triage tools

It is crucial to be able to predict how severe a patient’s medical condition is to support the early identification of those who are vulnerable and at high-risk, especially in emergency medical services. In a study, authors developed and validated an A.I.-based algorithm using deep learning that accurately predicted the need for the critical care of patients and outperformed the conventional triage tools and early warning scores^23^. In another study, authors analyzed online triage tools from more than 150 000 patient interactions with a chatbot, and they found a decreased level of the urgency of patients’ intended level of care in more than one-quarter of the cases^24^. Both studies indicate that A.I.-based technologies can facilitate triaging even before patients reach the point-of-care.

## How can an A.I.-based medical technology become part of an everyday practice?

The success of A.I. and its place in medicine and healthcare highly depend on whether it can penetrate the boundaries of evidence-based medicine, the lack of policies and reluctance from medical professionals to use it. There is no reason to believe that its use will be able to become common practice without meeting the standards and requirements of previous technologies.

However, as the demand for A.I. to be implemented into everyday medicine is getting higher by patients, policy makers, medical professionals and hospitals, its way from developers to practice will have to become faster^25^. A typical example of how it has worked so far is related to Kardia, formerly known as AliveCor.

They first made an FDA-approved smartphone case that worked as a single lead ECG in 2012. They launched two clinical trials to test the hardware and the app comparing it to a traditional 12-lead device. Later, the evolution of its design resulted in a credit-card sized device and an even smaller version in 2019. The original device could provide a one channel ECG by playing the user’s fingertips on the sensor for 30 s. The results were uploaded to the cloud to make it accessible for physicians. In 2015, Alivecor received clearance by the FDA to use an algorithm for the analysis of the readings to determine issues related to heart rhythm without human help.

By the end of 2017, they already used deep learning networks, and the FDA cleared the company’s ECG reader called KardiaBand as a medical device accessory to the Apple Watch. A study concluded that the device managed to distinguish between atrial fibrillation and a normal heart rhythm with a sensitivity of 93, and a specificity of 94%, respectively. Its sensitivity increased to 99% when a medical professional reviewed the reading^26^.

By 2020, products of Alivecor have been tested in over 40 clinical studies. Despite these accomplishments, the use of the device is still not common practice. And as other companies producing A.I.-based medical technologies are lagging behind, it might depict a long period of adoption.

A.I. will only reach the status of an everyday medical technology if medical associations provide clear guidelines about implementing it; if policy makers create policies that favor adoption; and if the medical community does not look at A.I. as a threat, but rather, as the stethoscope of the 21st century.

## What are the major challenges ahead?

Examples for ANI do exist today, but there are major issues the A.I. developer and the medical community have to face and tackle before A.I. can become mainstream in medicine.

### Explainability

Medical professionals tend to make decisions using data that were obtained with technologies they either understand or understand the basics enough to trust it. In the case of A.I., it might not be possible. However, millions of learned parameters (the connection weights within the network) determine the output of a deep neural network, which makes it unintuitive to understand the decision process. Even if we visualize the sensitivity of different parts of a network and browse these thousands of noisy images, we will still not see easy-to-grasp learned rules. Reasoning is not a by-product of the algorithm. Thus, explainable A.I. would be crucial in providing insights into A.I.-based algorithms enough to gain trust in them.

### Augmented intelligence

This is a term often promoted by organizations such as the American Medical Association. It focuses on A.I.’s assistive role in healthcare emphasizing that A.I.‘s design enhances human intelligence rather than replaces it. It also refers to the value an A.I. can provide that comes from how we can combine the unique capabilities of human experts with those of A.I. to provide better care for patients. A similar term that is related to augmented intelligence is “human-centered A.I.” which explores the need for the development of A.I.-based systems that learn from and collaborate with humans in a deep and meaningful way.

### Quality and quantity of data

A.I. feeds on data. The more and better quality data it gets access to, the more it can excel at tasks. Advanced algorithms need annotated data to make sure those can learn the task they were designed for. There are medical professionals who act as data annotators which is a time-consuming and monotonous task. Some medical algorithms can only improve through large amounts of annotated data. Therefore the dedicated contribution of data annotators is of crucial importance for the benefit of implementing A.I. in the healthcare setting. Therefore, we can conclude that data annotators are the unsung heroes of the medical A.I. revolution^27^.

### Privacy issues

Medical A.I. needs access to medical records, data from health sensors, medical algorithms, apps and whatever source of information it can learn from. The data can come from healthcare institutions or from individuals. Even if institutions make data anonymized, it was proven in many cases that individual profiles can be traced back.

### Legal issues and liability

What if a deep learning algorithm misses a diagnosis, the doctor accepts the judgment and the patient suffers from the consequences? What if an autonomous surgical robot injures a patient during a procedure? It is an ongoing debate about who will be held liable in the future when robots and A.I., acting autonomously, harm patients. Current consensus states that the professional is open to liability if he or she used the tool in a situation outside the scope of its regulatory approval, or misused it, or applied it despite significant professional doubts of the validity of the evidence surrounding the tool, or with knowledge of the toolmaker obfuscating negative facts. In any other cases, liability falls back on the creators and the companies behind them.

### Trust

We will need a lot of time to trust an autonomous car, to see how it reacts in situations we are familiar with or whether it makes similar decisions in an emergency. Consequently, it will take even more time not only for patients but for medical professionals too to trust A.I. with medical diagnoses, supporting medical decision-making or designing new drugs. This should be taken into consideration when we decide to adopt the technology into the healthcare setting.

### Biased A.I.

A study concluded that commercial companies’ facial-recognition systems were more accurate on lighter-skinned individuals by 11–19%. Those produced especially inaccurate results when identifying women of color. In another example, A.I. was implemented in the United States’ criminal justice system in order to predict recidivism. They found that the algorithm predicted disproportionately high probability of black people committing future crimes, no matter how minor their initial offense was. It is not only racial prejudice, but A.I. algorithms also often discriminate against women, minorities, other cultures, or ideologies. For example, Amazon’s HR department had to stop using their A.I.-based machine learning tool which the company developed for sorting out the best job applicants, as it turned out that the smart algorithm favored men. As such algorithms learn from the data they are fed with, A.I. programmers must know about the issue of bias in algorithms and actively fight against it by tailoring them^28^.

### Patient design

When designing algorithms for medical purposes, patients should be involved on the highest level of decision making to make sure their needs are met, and issues and recommendations are built into the technology. An example about its importance is how a start-up developed an algorithm that could detect signs of Alzheimer’s disease in phone calls of patients in Canada. However, it showed different results with patients who had a French accent. By inviting patients at the early stages of development, such issues could be avoided.

As there are positive ongoing efforts for solving each of these, it is still an open question whether those algorithms that become a common part of medical practices would be able to address them all^29^.

## The future role of A.I. in medicine and healthcar**e**

Every A.I.-based technology that is considered for use in healthcare must be regulated, efficient and backed by evidence^30^. The US Food and Drug Administration (FDA) has been showing an example in achieving a regulatory environment that not only welcomes such innovations but is also able to keep them safe for the public. The FDA launched a branch for digital health in 2019 and has attempted at designing new regulatory standards for A.I.-based technologies.

The FDA realized that in the era of A.I., more algorithms will become available than medical devices as there is a shift from hardware to software. As the number of algorithms to be regulated grows exponentially, the current resources of regulatory bodies will not be sufficient for assessing each iteration and update. There have been discussions around a new regulatory framework for modifications to A.I./machine learning-based software as a medical device (SaMD)^31^. This could potentially lead to regulations that make it possible for regulators to assess companies while the companies can roll out algorithms and updates without the need to check them all. This is a viable way of letting A.I.-based technologies become widespread while keeping them safe. Our research group has attempted at creating a constantly updated database of FDA-approved A.I.-based medical technologies. After cross-checking and validating every approval we could find, we identified 64 A.I.-based and, at the same time, FDA approved medical technologies. Only 29 of those (45%) mentioned any A.I.-related terms or expressions in the announcement released by the FDA.

Such technologies can only be efficient if they are successfully implemented into the medical practice. The American Medical Association (AMA) has addressed the importance of A.I., has advocated for the use of the expression augmented intelligence, and has assumed thought leadership with its reports and guidelines for physicians. In their AI policy, they state that “as a leader in American medicine, our AMA has a unique opportunity to ensure that the evolution of AI in medicine benefits patients, physicians and the health care community”^32^.

To make sure that A.I.-based technologies meet the standards of evidence-based medicine, numerous editorial boards of medical journals and prestigious medical associations such as the WHO or CDC have released their recommendations for the medical community^33,34^.

One of the potential obstacles in adoption can be a common fear among medical professionals that A.I. will replace them. While highly repetitive and data-based professions, or rather tasks under those professions will probably be highly impacted by automation, the core of the medical profession is and still will be the human touch, empathy and compassionate care; attributes that are almost impossible to mimic through a programming language.

However, as a general rule of thumb, we might be able to assume that those medical professionals who use A.I. will replace those who do not do so. That is how such a profound role A.I. will have in the future of medicine. The debate early in the 21st century should not be about whether A.I. takes away the human touch or the art of medicine, but what we should do to improve both with it.

When an algorithm using reinforcement learning, thus not being restricted by our human cognitive limitations, comes up with a cure which we could never find with our knowledge about biology, medicine and other life science, the real art of medicine will be to find out and to understand how it did that^35^.

With this basic knowledge about the definition, levels, methods, challenges and potentials of A.I., we tried to give an overview about how we can make the medical profession more creative, spending more time with patients than ever.
